# Cross-sectional associations of optimism with artery calcification and function: The SCAPIS study

**DOI:** 10.1177/22799036221110021

**Published:** 2022-09-28

**Authors:** Yvonne Natt och Dag, Gunnar Engström, Maria Rosvall

**Affiliations:** 1 School of Public Health and Community Medicine, Institute of Medicine, Sahlgrenska Academy at University of Gothenburg, Gothenburg, Sweden; 2 Department of Clinical Sciences in Malmö, Lund University, Malmö, Sweden; 3 Social Medicine, Primary Health Care, Regionhälsan, Region Västra Götaland, Gothenburg, Sweden

**Keywords:** Optimism, psychosocial, atherosclerosis, epidemiology

## Abstract

**Background:**

An increasing amount of research indicates that positive psychological factors, such as optimism, might be beneficial for cardiovascular health. However, most studies have focused on cardiovascular events. The present study aimed to investigate associations between optimism and subclinical outcomes related to cardiovascular health.

**Methods:**

This cross-sectional study used data from SCAPIS Malmö, Sweden, including 6251 randomly selected men and women from the Malmö municipality area, aged 50 to 64 years. Optimism was assessed via the LOT-R questionnaire, but also by using the two subscales of LOT-R, assessing optimism and pessimism separately. Arterial health was assessed as the coronary artery calcium score, ankle-brachial index, and aortic augmentation index. Cardiovascular risk was estimated using the SCORE instrument. Adjustments were made for sociodemographic factors, depression, and cardiovascular risk factors.

**Results:**

Those who were most optimistic had lower odds of coronary artery calcification, with an odds ratio of 0.74 (95% confidence interval 0.58, 0.93), compared to those who were least optimistic. Also, higher levels of optimism were associated with a general pattern of lower aortic augmentation index, and with higher ankle-brachial index on both left and right side. For coronary artery calcification associations seemed to be mediated primarily through an absence of pessimism. The associations were reduced after adjustments, but persisted for measures of arterial function.

**Conclusions:**

The results indicate that optimism might be health protective with regard to arterial function, but with regard to coronary artery calcification it was rather the absence of pessimism that was of importance.

## Background

In recent years, there have been increasing ambitions to go beyond the goal of simply reducing risk of disease, to also promote healthy life and longevity.^1^ While psychosocial distress factors such as depression or negative life events have been associated with increased risk of cardiovascular morbidity and mortality,^2,3^ less is known about the potential protective effects of a positive mindset such as dispositional optimism. Dispositional optimism is most often defined as having a bright vision of the future and expecting good things to occur.^4^ Optimists have been shown to be more likely to succeed in relationships, and to have more favourable health behaviours, problem-solving capacity, and stress-resilience, compared to their less optimistic peers.^4–7^ Despite its relative stability, evidence indicates that optimism may vary with life transitions as well as with short-term challenges, and that it is also modifiable by interventions.^7–10^

In a recent review and meta-analysis, including 15 studies, participants with a more optimistic mindset were at lower risk for cardiovascular events and mortality^4^; the authors of the review suggested that future studies should seek to evaluate the bio-behavioural mechanisms involved in the associations between optimism and health. Studies investigating associations between optimism and cardiovascular health markers have reported that optimistic persons have reduced risk of hypertension^9^ and decreased progression of carotid atherosclerosis.^11^ Optimism may be an important attribute for maintenance of cardiovascular health in early middle age.^12^ However, there are also negative findings.^13–15^ Optimism is easy to measure; the most frequently used instrument is the well-validated Life Orientation Test-Revised (LOT-R).^16^ To the best of our knowledge, no previous study has connected optimism to both coronary calcification and arterial function. It is important to elucidate these associations in order to better understand potential protective effects early in the disease process, not least in view of the increasing prevalences of obesity and cardiovascular morbidity among the young in many countries.^17^

The present study aimed to investigate associations between LOT-R and arterial health, assessed by way of non-invasive measurements of aortic augmentation index (aortic AIx), ankle-brachial index (ABI), and coronary artery calcium score (CACS), respectively.

## Design and methods

### Study population

The Swedish CArdioPulmonary bioImage Study (SCAPIS) is a Swedish population-based cross-sectional study performed at six university hospitals (*n* = 30,154 participants), with the main aim of early detection of cardiovascular and lung disease.^18^ The same study protocol was used for all study sites, although with possibilities to add optional examinations depending on local research interests. The present study used data from the SCAPIS Malmö cohort (*n* = 6251; participation rate 53%), comprising randomly selected men and women aged 50–64 years from the Malmö municipality area. There were no exclusion criteria, except for inability to understand written and spoken Swedish for informed consent.^18^ Inclusions took place 2014–2018, with extensive assessments over 3 days: the participants answered questionnaires; clinical examinations and physiological measurements were performed, and blood tests were taken. The Malmö cohort were asked questions on optimism in addition to the core questions in SCAPIS. The study was approved by the Ethical Committee (Dnr 2016/1031 and 2020-02746). Participants with missing information on optimism (*n* = 837) were excluded from the analyses, and the remaining 5414 subjects constituted the study population. The excluded subjects had similar mean age (57.5 vs 57.5 years), proportion of men (45.2% vs 47.1%) and proportion with coronary artery calcification score (CACS) ≥100 (15.2% vs 13.7%) as those included in the study.

### Coronary calcium score, augmentation index, and ankle-brachial index

Coronary artery calcification was assessed in non-contrast-enhanced images from a state-of-the-art multi-slice computed tomography scanner (Somatom Definition Flash, Siemens Healthineers, Erlangen, Germany). Imaging and analyses were performed using a calcium scoring protocol, and the calcium content in each coronary artery was measured and summed to produce a total coronary artery calcification score (CACS) in Agatston units.^19,20^ Ankle-brachial index (ABI) was measured bilaterally using a doppler pulse sensor, and was calculated as the highest systolic mean blood pressure of *a dorsalis pedis* or *a tibialis posterior*, divided by the average of two supine brachial blood pressures, in the arm with the highest blood pressures. The aortic augmentation index (AIx) was used to determine arterial stiffness, and was measured with the SphygmoCor XCEL applanation tonometry device (Atcor Medical, Australia). Prior to the examination, participants were instructed to refrain from caffeine and heavy meals for 3 h, nicotine for 4 h, and alcohol for 12 h. Blood pressure cuffs were attached to the upper left arm and to the right thigh (10–20 cm below the groyne). AIx was standardised to a heart rate of 75 bpm.

### Life-orientation test revised (LOT-R)

Optimism was assessed with the LOT-R scale. The scale comprises 10 items, including four filler items. Three of the six scored items are negatively worded, reflecting pessimistic attitudes, and the remaining three are positively worded, reflecting optimistic attitudes. Responses were rated 0–4 on a Likert scale, where 0 denoted ‘strongly disagree’, and 4 denoted ‘strongly agree’. Items with a negative wording were reverse coded before scoring, and thus higher scores indicated greater optimism (range 0–24). In addition, for purpose of secondary analyses, the three negatively worded questions in LOT-R were summed into a pessimist subscale (range 0–12), and the three positively worded questions were summed into an optimist subscale (range 0–12).^21^ The Swedish translation of LOT-R was used.^22^

### Sociodemographic factors and depression

Educational attainment was categorised as presence or absence of a university or college degree. Marital status was categorised as married or cohabiting, versus living alone. Depression was assessed by the question: ‘During the past 12 months, have you ever felt sad, blue, or depressed, for a period of 2 weeks or more?’; a positive answer was further explored by seven additional yes/no questions: whether during such a period the respondent had lost interest in most activities, felt tired and lacked energy, had gained/lost weight, had experienced more difficulties falling asleep, had trouble concentrating, had many thoughts about death, or felt worthless. Depression within the last 12 months was defined by the answer ‘yes’ on five or more of these questions. These questions are adapted from the CIDI-SF questionnaire for depression,^23^ and have been used in the INTERHEART study.^24^

### Cardiovascular risk factors

Smoking status was categorised as ‘current smoker’ or ‘non-smoker’. Leisure-time physical activity was assessed through questionnaire and dichotomised into ‘sedentary’ (mainly sitting: reading, TV, at the computer) or ‘not sedentary’ (moderate, regular, or vigorous exercise). Alcohol consumption was assessed in tertiles based on the frequency of intake of different types of alcoholic beverages measured using the MiniMeal-Q questionnaire and converted into grams of ethanol consumed per day.^25^ Abstainers were added to the first tertile. Intake of saturated fat (g/day) was also assessed through the MiniMeal-Q questionnaire.^25^ SCORE was calculated from sex, age, smoking, systolic blood pressure and total cholesterol, and categorised into low risk (<1%), moderate risk (1–4%), high risk (5–9%), and very high risk (≥10%).^26^ Participants with established CVD (i.e. history of acute coronary syndrome, percutaneous coronary intervention, coronary artery bypass grafting, stroke, or arterial stenosis treatment), diabetes, or high cholesterol were always categorised as very high risk, regardless of score. Body weight and height were measured with participants in light clothing and without shoes, and body mass index (BMI) was calculated as weight in kg divided by square of height in m. Diabetes was defined as glucose ≥7.0 mmol/l and/or HbA1c ≥ 48 mmol/mol, or previously known diabetes. Individuals with glucose ≥7.0 mmol/l at the examination were rescheduled for a repeated blood glucose test to confirm the diagnosis. Systolic blood pressure was measured twice in both arms with the participant in a lying position after a 10-min rest. The mean blood pressure in the arm with the highest pressure was used.

### Statistical methods

Subjects with missing data on LOT-R were excluded from the study population. LOT-R scores were divided into quartiles, and categorised into three groups: least optimistic (first quartile), moderately optimistic (second and third quartiles), and most optimistic (fourth quartile), using the first quartile of optimism as the reference level, for the descriptive analyses and logistic regression models. The choice of using quartiles was based on the fact that there are no evident cut-offs for the LOT-R. Differences in prevalences and means of sociodemographic factors, life style factors, depression, and cardiovascular risk factors, with regard to quartiles of LOT-R, were analysed with chi-square and F-tests (Table 1). Since CACS was not normally distributed, this variable was categorised into having CACS ≥ 100 or not. This cut-off value has in previous studies been assessed as clinically relevant and associated with highly increased risk of CVD.^27^ Furthermore, the number of coronary vessels with presence of calcification (i.e. left main and anterior descending artery (LAD-LM), circumflex artery (CIRC), and/or right coronary artery (RCA) with scores >0) were categorised into 2 or 3 versus 0 or 1. Logistic regression analyses were performed to calculate age- and sex-adjusted, and multiply-adjusted odds ratios (ORs) with 95% confidence interval (95% CI) (Table 2). Aortic AIx and ABI were analysed as continuous variables. Linear regression analyses were performed to calculate age- and sex-adjusted, and multiply-adjusted models with 95% CI per 10-units increase in LOT-R (Table 3). Adjustments for covariates in the multiply adjusted models were made in four models. Missing data on included covariates constituted at most about 2% of the study population. Model 1 made adjustments for age and sex, and Models 2–4 each comprised Model 1 with additional adjustments: Model 2 for marital status and educational level; Model 3 for depression; and Model 4 for SCORE categories, alcohol consumption, BMI, saturated fat intake, physical activity, and antihypertensive and antilipid treatment. Finally, a multiple adjustment model was applied, including all these covariates. Furthermore, secondary analyses were performed excluding subjects with prevalent CVD. Secondary analyses were also performed using the two subscales of LOT-R, that is, pessimism (range 0–12) and optimism (range 0–12).

**Table 1.** Distribution as mean (standard deviation) or percent of sociodemographic, lifestyle, depression, and biological risk factors by quartiles of optimism measured through Life orientation test—Revised (LOT-R).

	Least optimistic	Moderately optimistic	Most optimistic	*p*-Value
	First quartile (*n* = 1417)	Second and third quartiles (*n* = 2641)	Fourth quartile (*n* = 1356)	
Age	57.4 (4.2)	57.5 (4.3)	57.6 (4.3)	0.59
Male sex	44.1	49.2	46.2	0.006
University education	33.1	43.4	50.0	<0.001
Married or cohabiting	60.9	72.7	74.3	<0.001
Current smoking	23.2	15.2	14.2	<0.001
Highest tertile of alcohol consumption	28.5	36.8	36.9	<0.001
Low physical activity	22.8	13.7	8.2	<0.001
Saturated fat (g)	30.1 (23.3)	27.6 (20.2)	25.7 (15.6)	<0.001
BMI	27.7 (5.0)	27.2 (4.5)	26.7 (4.3)	<0.001
Systolic blood pressure	124.8 (16.8)	123.1 (16.3)	123.4 (17.4)	0.005
Diabetes	10.9	7.1	6.7	<0.001
Score				
Low <1%	20.4	21.0	23.3	
Medium/high 1%–9%	64.0	67.7	66.5	
Very high 10%	15.6	11.4	10.1	<0.001
Depression	35.5	14.4	6.7	<0.001

**Table 2.** Coronary artery calcium score (CACS) by quartiles of optimism measured through Life orientation test—Revised (LOT-R).

	Least optimistic	Moderately optimistic	Most optimistic	*p* for trend
	First quartile	Second and third quartile	Fourth quartile	
CACS ≥ 100 (Yes vs No)				
Odds ratio (95% confidence interval)				
Adjustments				
Model 1^a^	1.00	0.82 (0.68, 0.99)	0.74 (0.58, 0.93)	0.009
Model 2^b^	1.00	0.87 (0.72, 1.06)	0.80 (0.63, 1.00)	0.055
Model 3^c^	1.00	0.83 (0.68, 1.00)	0.74 (0.58, 0.93)	0.013
Model 4^d^	1.00	0.90 (0.73, 1.10)	0.84 (0.66, 1.07)	0.15
Multiply adjusted model^e^	1.00	0.92 (0.74, 1.13)	0.87 (0.67, 1.11)	0.26
Number of vessels with coronary artery calcification (2–3 vs 0–1)				
Odds ratio (95% confidence interval)				
Adjustments				
Model 1^a^	1.00	0.71 (0.60, 0.84)	0.71 (0.60, 0.87)	<0.001
Model 2^b^	1.00	0.75 (0.63, 0.88)	0.77 (0.64, 0.94)	0.007
Model 3^c^	1.00	0.72 (0.61, 0.85)	0.73 (0.60, 0.89)	0.002
Model 4^d^	1.00	0.78 (0.66, 0.93)	0.82 (0.68, 1.00)	0.054
Multiply adjusted model^e^	1.00	0.80 (0.68, 0.95)	0.86 (0.70, 1.05)	0.14

a Adjusted for age and sex.

b Model 1 with additional adjustment for university education, and being married/cohabiting.

c Model 1 with additional adjustment for depression.

d Model 1 with additional adjustment for SCORE categories, BMI, alcohol consumption, intake of saturated fat, low physical activity, antihypertensive medication, and antilipid medication.

e Adjusted for model 1–4.

**Table 3.** Regression coefficients and 95% confidence interval of aortic AIx and ankle-brachial index by increase (per 10-units) in optimism measured through Life orientation test—Revised (LOT—R).

	Aortic AIx 75 beats/min	Ankle-brachial index (left)	Ankle-brachial index (right)
	*b* (95% CI)	*b* (95% CI)	*b* (95% CI)
Model 1^a^	−2.50 (−3.20, −1.79)	0.013 (0.007, 0.019)	0.015 (0.009, 0.021)
Model 2^b^	−2.11 (−2.83, −1.41)	0.011 (0.005, 0.016)	0.013 (0.007, 0.019)
Model 3^c^	−2.22 (−2.96, −1.49)	0.013 (0.007, 0.019)	0.016 (0.010, 0.023)
Model 4^d^	−1.30 (−1.97, −0.59)	0.007 (0.001, 0.013)	0.009 (0.003, 0.016)
Multiply adjusted model^e^	−1.06 (−1.79, −0.33)	0.006 (0.001, 0.013)	0.010 (0.003, 0.015)

a Adjusted for age and sex.

b Model 1 with additional adjustment for university education, and being married/cohabiting.

c Model 1 with additional adjustment for depression.

d Model 1 with additional adjustment for SCORE categories, BMI, alcohol consumption, intake of saturated fat, low physical activity, antihypertensive medication, and antilipid medication.

e Adjusted for model 1–4.

## Results

Table 1 shows the study population by sociodemographic and risk factor characteristics, with respect to LOT-R score quartiles. There were no differences in mean age between the quartiles. Individuals with a university education, those who were male, and those who were married or cohabiting, more often scored high on the LOT-R, while individuals with depression, current smoking, low physical activity, diabetes, high systolic blood pressure, and high intake of saturated fat, more often scored low. Individuals with high alcohol consumption were less likely to score in the lowest tertile.

Information on CACS was obtained for 5242 participants. Table 2 shows the adjusted associations between optimism and CACS. Those with higher levels of optimism had lower odds of increased CACS, as well as lower odds of having multiple coronary vessels affected by coronary calcification. These trends generally were only slightly attenuated after adjustment for sociodemographic factors and depression, respectively, but lost statistical significance after adjustment for cardiovascular risk factors. Secondary analyses excluding participants with prevalent CVD (*n* = 135; 2.6%), did not change the found patterns of associations (data not shown). Secondary analyses using the pessimism subscale demonstrated consistent significant associations (*p* for trend <0.05) with increased CACS, as well as with number of vessels affected by coronary calcification, whereas there were weaker and non-significant associations when using the optimism subscale (data not shown).

Information on aortic AIx was obtained for 5305 participants, while 5395 had information on ABI (left) and 5394 on ABI (right). The mean (SD) aortic AIx, ABI (left), and ABI (right) was 21.8 (11.7), 1.24 (0.09), and 1.25 (0.10), respectively. Table 3 shows regression coefficients (with 95% CI) of aortic AIx, and left and right ABI, by levels of optimism, as per 10 units of LOT-R. There was a consistent pattern of lower aortic AIx and higher ABI on both left and right side, with increasing optimism. These associations persisted in the fully-adjusted models, and exclusion of participants with an ABI > 1.40 did not change these results (data not shown). Neither did excluding participants with prevalent CVD change the found patterns of associations, except for a weakening of the association between LOT-R categories and ABI left in model 4 and in the multiply adjusted (final) model (data not shown). Secondary analyses using the two subscales of LOT-R showed significant associations (*p* for trend <0.05) between pessimism and ABI right, ABI left, and aortic Aix, respectively; for optimism, there were significant associations with ABI left and aortic Aix. These associations persisted for pessimism, but not for optimism, in the fully adjusted models (data not shown).

## Discussion

In this population-based sample aged 50–64 years, cross-sectional associations were observed between higher LOT-R scores and reduced odds of increased CACS. The associations generally remained significant after adjustment for sociodemographic factors and depression, respectively, but lost significance after adjustment for cardiovascular risk factors. Higher LOT-R scores were also associated with reduced heart-rate-corrected augmentation index, and increased ankle-brachial index; these associations persisted in the fully-adjusted models. Secondary analyses on the LOT-R subscales revealed that the associations between LOT-R scores and CACS were primarily due to absence of pessimism, rather than to presence of optimism.

Few studies have investigated associations between optimism and arterial health and function.^11,13–15^ One of these was performed on a smaller population-based sample of middle-aged women, and showed decreased progression of carotid atherosclerosis among those who were more optimistic, even after adjustment for biological and life-style factors.^11^ However, another study found no associations between optimism and progression of CAC.^13^ In a third study, on a small community-based sample of men and women, no cross-sectional associations were found between optimism and carotid intima media thickness.^14^ A fourth study, comprising 1849 participants, found no cross-sectional associations between optimism and CAC; the authors pointed out that risk factor chronicity needs to be further investigated in relation to development of CAC.^15^ Research on optimism in relation to clinical cardiovascular outcomes is more extensive; a meta-analysis including 15 studies recently concluded that optimism was associated with reduced risk of cardiovascular morbidity and mortality. The associations generally persisted after adjustments for sociodemographic factors and depression; however, most studies did not have data on major cardiovascular risk factors. The authors of the meta-analysis concluded that further studies are needed to better define the mechanisms involved.^4^

It has been argued that, in addition to using the overall score of the LOT-R, the positively and negatively worded questions (assessing optimism and pessimism, respectively) should be separated and used as subscales, since optimism and pessimism might be partly distinct constructs.^14,16,28–30^ In the present study, results from secondary analyses using the optimism/pessimism subscales indicate that the associations between LOT-R scores and CAC are mediated through an absence of pessimism, rather than through presence of optimism. Similar findings were seen in a study on cardiovascular mortality, where only pessimism, but not optimism, was associated with CVD mortality.^21^ The findings are in accordance with a meta-analysis on optimism and physical health, where it was observed that the mean effect sizes of pessimism were larger than the mean effect sizes of optimism, although not significantly so.^28^ A recent meta-analytic analysis of data from earlier studies, however, has demonstrated significantly higher effect sizes of pessimism as compared to optimism, when aggregating various physical outcomes.^29^ In the present study, there were evident associations of both optimism and pessimism with arterial function, indicating that, for these outcomes, both parts of the LOT-R construct may be of relevance. However, an earlier study, examining cross-sectional associations between LOT-R scores and mean arterial pressure (MAP) in a community sample of 300 men and women, found that lower pessimism, but not higher optimism, was associated with nocturnal dipping of MAP, which is considered an indicator of healthy arterial function.^14^ Other data suggest that pessimism but not optimism is independently associated with inflammation.^7^ It could tentatively be suggested that optimism and pessimism are differentially associated with physical health, but more research is needed for elucidation.^29,30^

Conceptually, the effect of optimism on physical health might be explained by favourable health-related behaviour, by presence of psychosocial resources, and/or by direct biological effects due to inhibition and/or buffering of stress responses.^1,7^ Indeed, optimists have been shown to adopt health-promoting behaviours such as exercising, healthy eating, and avoiding smoking,^7,31^ and also to hold better problem-solving capacity and stress-resilience,^5–8^ compared to less optimistic individuals. It has been argued that, since many cardiovascular risk behaviours, such as eating habits and physical activity, are difficult to change, it might be necessary to address upstream psychological factors, such as optimism, in order to promote healthy behaviour.^9^ The possibility of initiating and promoting virtuous circles of healthy behaviour, by addressing maladaptive coping strategies, has also been discussed.^7^ Such strategies would conceivably be of particular relevance with respect to the increasing cardiovascular morbidity among the young.^17^

In the present study, individuals who were more optimistic had a more favourable socioeconomic position and better health-related behaviours, and were less likely to report depression. Previous studies have shown that more optimistic persons less often experience depression or depressive symptoms,^6^ and social isolation.^7^ In the present study, the associations with CACS lost statistical significance in the fully-adjusted models, suggesting that the effect of optimism works through the covariates adjusted for, and predominantly so through cardiovascular risk factors. However, although the associations with arterial function were reduced, these associations persisted, implying that additional factors might be of importance.

All cardiovascular outcome measures used in the present study (CACS, ABI, and aortic AIx), have been demonstrated to be markers of cardiovascular risk, and to predict future cardiovascular events.^15,27,32–35^ The differences in ABI and aortic AIx by increase in LOT-R scores were rather small, but might still be clinically relevant. Previous studies have reported increases in cardiovascular risk even with smaller changes in arterial anatomy and function. For example, in a large German population-based study on middle-aged men and women, the hazard ratio for incident major cardiovascular event per 1 SD increase in ABI (SD 0.14) was 1.37 (95% CI 1.20, 1.55).^34^ A meta-analysis showed that AIx increased the risk of cardiovascular events and all-cause mortality, with hazard ratios of 1.32 (95% CI 1.09, 1.59) and 1.38 (95% CI 1.19, 1.61), respectively, per absolute 10% increase in aortic AIx.^35^ Moreover, regarding the predictive value of CAC, a recent pooled analysis of data from Multi-Ethnic Study of Atherosclerosis (MESA) and Dallas Heart Study (DHS) demonstrated a three- to five-fold increased risk of coronary heart events, and a two- to three-fold increased risk of cardiovascular events, associated with CACS ≥ 100, over a follow-up of 10 years.^27^ Also, it has been pointed out that even small effect sizes can be of considerable relevance for public health, which is dealing with entire populations comprising large numbers of individuals.^29^

The primary strengths of the present study are the large population-based sample, the relatively high response rate, the extensive information on cardiovascular risk factors, and the use of non-invasive measures as markers of early cardiovascular disease. Furthermore, the exposure assessment instrument LOT-R has been much used in research and has demonstrated both validity and reliability.^16^ In the present study, the items included in the two subscales on optimism and pessimism, respectively, showed good internal consistency (data not shown). There are also some methodological limitations. First, the cross-sectional design does not provide any information on causation, and so it is possible that presence of arterial changes causes people to report less optimism. Early arterial changes are presumably asymptomatic, but again, the possibility of reverse causation cannot be ruled out. It is noteworthy, however, that in a recent meta-analytic analysis of data from earlier studies on optimism and pessimism, the effect sizes in cross-sectional and prospective studies were comparable.^29^ A second limitation is that a rather large part of the original sample (about 800 individuals) had missing information on LOT-R. Individuals with missing LOT-R data, however, had similar mean values on aortic AIx, and on ABI left and right, respectively, as those included in the analyses, and a similar proportion had CACS ≥ 100 (data not shown). These similarities in arterial health (i.e. between individuals with and without LOT-R data) suggest that LOT-R data were mainly missing at random.

## Conclusions

Cross-sectional associations were found between optimism and healthier arteries, measured with three different non-invasive methods. The results indicate that optimism might be health protective with regard to arterial function, but with regard to coronary artery calcification it was rather the absence of pessimism than presence of optimism that seemed to be of importance. Pessimism may be related with maladaptive coping strategies inducing toxic stress responses and inflammation, as well as detrimental health behaviours, while optimism may be an important asset for obtaining cardiovascular health. The biobehavioural mechanisms need to be further elucidated, and further knowledge on how to address pessimism and how to promote optimism is warranted.
